# Analysis of the Association between Fat Mass Distribution and Bone Mass in Chinese Male Adolescents at Different Stages of Puberty

**DOI:** 10.3390/nu13072163

**Published:** 2021-06-24

**Authors:** Kai-Li Deng, Hui Li, Wan-Yu Yang, Jin-Li Hou, Yang Xu, Su-Mei Xiao

**Affiliations:** 1 Department of Epidemiology, School of Public Health, Sun Yat-sen University, Guangzhou 510080, China; dengkli@mail2.sysu.edu.cn (K.-L.D.); lihui256@mail2.sysu.edu.cn (H.L.); yangwy29@mail2.sysu.edu.cn (W.-Y.Y.); houjli@mail2.sysu.edu.cn (J.-L.H.); xuyang58@mail2.sysu.edu.cn (Y.X.); 2 Guangdong Provincial Key Laboratory of Food, Nutrition and Health, School of Public Health, Sun Yat-sen University, Guangzhou 510080, China

**Keywords:** fat mass, fat mass distribution, bone mass, adolescent, puberty

## Abstract

Background: Bone mineral acquisition during adolescence is crucial for maximizing peak bone mass. Fat mass (FM) and bone mass are closely related. This study investigated the association of FM distribution with bone mass in Chinese male adolescents. Method: A total of 693 male adolescents aged 10–18 years were recruited from a secondary school in Jiangmen, China. Their bone mass and body composition were measured by quantitative ultrasound and bioelectrical impedance analysis, respectively. The associations of the measures of fat distribution with bone parameters, i.e., broadband ultrasound attenuation, speed of sound (SOS), and stiffness index (SI), were analyzed using multiple linear regression. Age, height, body mass index, stage of puberty, physical activity, sedentary behavior, dietary energy intake, and dietary calcium and vitamin D intake were adjusted in the model. Further subgroup analyses of prepubertal and pubertal participants were conducted. Results: The measures of fat distribution showed negative associations with SOS and SI in total subjects (*p* < 0.010). In prepubertal boys, the measures of fat distribution were only associated with SOS (β = −0.377 to −0.393, *p* < 0.050). In pubertal boys, the measures of fat distribution had associations with all bone parameters (β = −0.205 to −0.584, *p* < 0.050). The strongest association was between trunk FM and SOS (β = −0.584, *p* < 0.001). Conclusion: This study supported that the measures of fat distribution were negatively associated with bone parameters in Chinese male adolescents. Trunk FM had the strongest association with bone parameter. These associations appear to be stronger in pubertal boys than in prepubertal boys.

## 1. Introduction

Osteoporosis is a common disease in both female and male populations. Previous studies have mainly focused on females, while more attention should also be paid to males. Studies have indicated that one in four men over 50 suffer an osteoporosis-related fracture in his lifetime [1]. Data from a systematic review and meta-analysis of 33 studies revealed that the prevalence of osteoporosis in Chinese elderly men was 23% [2]. Peak bone mass is a major determinant of the risk of osteoporosis in later life [3,4]. Adolescence is a crucial period for bone mineral acquisition. Bone mass increases by approximately 45% during puberty and reaches approximately 90% of its adult peak value by the end of puberty [5]. Obtaining sufficient bone mass during adolescence may therefore be a key factor in preventing osteoporosis.

Fat mass (FM) and bone mass are closely related [1,6]. However, accumulating evidence indicates that not all adipose tissue is equivalent. Studies in adults have found that adipose tissue in different parts of the body may have different effects on bone health [7,8]. A study of 686 individuals aged 20–39 years found that bone mineral density (BMD) decreased with central fat accumulation and increased with FM and percent body fat [7]. Another study of 1147 subjects, including Chinese, black, and white adults, revealed that percent body fat was negatively associated with BMD, and the percent of trunk fat to body weight exhibited a stronger relationship with BMD than did percent body fat [8]. Liu et al., in a study of 2465 middle-aged Chinese adults, found that limb fat in men and abdominal fat in women had the greatest unfavorable connection with BMD [9]. It is well known that abdominal fat distribution is a significant risk factor for metabolic diseases such as cardiovascular disease and metabolic syndrome [10,11]. It can therefore be conjectured that compared with total body fat, the pattern of fat distribution may play a more important role in bone health.

In adolescents, few studies have explored the influence of the distribution of fat tissue on bone mass. Most studies of the correlation of adipose tissue with adolescent bone mass have focused on the total body adipose tissue. Moreover, the results of those studies are inconsistent. Some studies have reported a positive association between total body FM and BMD or bone mineral content, whereas others have reported a negative or null association [12,13,14,15,16,17]. This disparity among studies could be due to the complex influence of FM in various parts of the body on bone health. The current consensus is that abdominal fat accumulation increases the risk of related metabolic complications, whereas gynoid fat deposition is associated with a decreased risk of related health problems [18]. Russell et al. found that visceral adipose tissue was negatively associated and subcutaneous adipose tissue was positively associated with BMD in female adolescents aged 12–18 years [19]. Among 710 adolescents aged 10–19 years who participated in the Korea National Health and Nutrition Examination Survey, the negative association between the percent of trunk fat and BMD was consistent across age groups, whereas the correlation of percent limb fat with BMD varied by age [20]. The regional FM distribution may have a higher predictive value than the total body FM for the assessment of bone health in adolescents. Moreover, puberty is one of the most critical periods for general fat development and its distribution in the body [21]. The change of adipose tissue distribution pattern during the puberty period could lead to the differences of the association between adipose tissue and bone parameters at different pubertal stages. But few studies have explored this aspect in adolescents.

It remains unclear how the distribution of FM contributes to bone mass accretion in adolescents and whether it is varied at different pubertal stages. In this study, we investigated the relationship between FM distribution and bone mass in Chinese male adolescents, and further detected the differences in this association at different stages of puberty.

## 2. Materials and Methods

### 2.1. Study Subjects

The subjects and data were from a cross-sectional study designed to investigate the musculoskeletal health in Chinese adolescents. Male students aged between 10 and 18 years from a secondary school in Jiangmen, China, participated in the survey voluntarily. The recruitment was carried out through posting advertisements and inviting students in each class at the school to attend health talks offered by the investigators. Individuals with disorders or taking medications that may affect bone or mineral metabolism, such as cancer, chronic kidney disease, or hypogonadism or steroid use, were ineligible. No students were excluded due to these criteria. Altogether, 745 boy students were initially invited to attend this survey between October and November 2015. Finally, 693 male participants were included in this study. The participate rate of this survey was 93.0%. The study was approved by the Ethics Committee of Jiangmen Central Hospital, Affiliated Jiangmen Hospital of Sun Yat-sen University, and informed consent was obtained from all study participants and their guardians.

### 2.2. Calcaneal Quantitative Ultrasound Measurements

Bone mass was tested by quantitative ultrasound (QUS), a method of estimating bone status by measuring the velocity and attenuation of ultrasound. QUS is particularly adaptable to evaluating bone mass in epidemiological studies with a relatively large number of healthy children and adolescents, due to its portable, low-cost, and radiation-free advantages, compared to the gold standard method of dual-energy X-ray absorptiometry (DXA) [22,23,24]. Studies revealed that the calcaneal QUS is a useful surrogate method for bone mass assessment and has a moderate to excellent correlation (r = 0.60–0.84) with DXA in the pediatric population [25,26]. QUS measurements at the calcaneus of the right heel were conducted with a Sahara Clinical Bone Sonometer (Hologic, Bedford, MA, USA). Three parameters—broadband ultrasound attenuation (BUA, dB/MHz), speed of sound (SOS, m/s) and stiffness index (SI)—were determined from the QUS measurements. BUA indicates bone mineral content. SOS is a reflection of bone microarchitecture and elasticity [27]. SI is calculated from the values of BUA and SOS using the following formula: SI = (0.67 × BUA + 0.28 × SOS) − 420. All measurements were performed by the same trained investigators. Quality control of the sonometer was conducted before the measurements by the direct apposition of a phantom signal into its transducers. The in vivo coefficients of variation for BUA and SOS were 2.3% and 0.2%, respectively.

### 2.3. Body Composition Measurements

Total body and regional FM and lean mass were measured by bioelectrical impedance analysis (BIA, InBody230, Korea Biospace Corporation, Seoul, Korea) with 10 impedance measurements each at two frequencies (20 and 100 kHz). BIA estimates body composition from resistance measurement of body tissues to an electric current. The human body is divided into five cylinders, i.e., the trunk, upper and lower extremities, with a uniform electric conductivity [28]. The measurements were performed in the morning with the participant in a fasting state with an empty bladder and not having drunk any liquid in the past 30 min. While measuring and recording total body and regional FM and lean mass (i.e., limbs and trunk regions), the participants were asked to wear light clothing, stand barefoot on the measurement panel after removing all metal objects and accessories and make their toes and heels fully in contact with the anterior and posterior electrodes. The measurements began when the grips were grasped by both hands. All of the tests were conducted by the same investigator following standard procedures. The precision was 1.0% and 0.3% for total body FM and lean mass, respectively. BIA is indicated as a good choice for assessing body composition because of its portability, low cost, and that it is easy to use. The correlation coefficients between BIA and the more accurate apparatus, i.e., magnetic resonance imaging (MRI) and DXA, for both total and regional FM were satisfactory (r > 0.80) in children and adolescents [29,30,31].

### 2.4. Evaluation of Covariates

A structured questionnaire was used to collect information on demographic characteristics, lifestyle habits, and histories of disease and medication use through face-to-face interviews implemented by trained staff. Dietary intakes of calories, calcium, and vitamin D were evaluated using the 24-h dietary recall record for 3 consecutive days, including meals and snacks [32,33]. It is one of the most commonly used dietary survey methods with good validity in adolescents [34,35,36]. The food pictures were used to help record the type and amount of food. The dietary intakes of calories and calcium were evaluated using the Chinese Food Composition Table [37]. The dietary intake of vitamin D was calculated using the Standard Tables of Food Composition in Japan [38]. The modified Chinese version of the Children’s Leisure Activities Study Survey questionnaire was used to assess physical activity and sedentary behavior [39]. It included 24 kinds of physical activity items above medium intensity such as football, volleyball, basketball, badminton, long jumping, etc., and 8 kinds of sedentary activity items, i.e., doing homework, TV viewing, online games. Then physical activity was expressed as metabolic equivalent (MET· h/d) after excluding low impact activities for bone such as swimming and cycling [40,41]. The sedentary behavior was expressed as hours per day. The Chinese version of the self-reported Pubertal Development Scale was adopted to evaluate the development stage of puberty [42]. This scale consists of five items: growth spurt, facial hair growth, body hair growth, deepening voice, and skin change. The participants were classified as prepubertal (stage I-II) (246 participants) and pubertal (stage III-V) (447 participants) using these data. Height was measured using a stadiometer to the nearest 0.1cm. Body weight was recorded in light clothing to the nearest 0.1 kg by Inbody 230 (Korea Biospace corporation, Seoul, Korea). Body mass index (BMI) was calculated as the weight in kilograms divided by the height in meters squared.

### 2.5. Statistical Analysis

The variables of the basic characteristics of the subjects were expressed as means and standard deviations (SD). Multiple linear regression analysis was used to test the correlations of total body and regional FM with bone parameters. In each model, a bone parameter value (i.e., BUA, SOS or SI) was the dependent variable, and total body FM, percentage of total body FM (total body FM%), trunk FM, limb FM, trunk-to-limb FM ratio, or total body FM-to-lean mass ratio were the independent variables. Subgroup analyses of prepubertal and pubertal participants were also conducted. Confounding factors were selected based on the previous literatures and the results of univariate analysis. The included confounders were age, height, BMI, physical activity, sedentary behavior, dietary energy intake, energy-adjusted dietary intakes of calcium and vitamin D, and stage of puberty (except for the subgroup analysis). There was collinearity between total body FM, trunk FM, limb FM, and body weight in this study (VIF > 10.0). BMI z-score instead of body weight was adjusted in the model. BMI z-score was calculated according to the age- and gender-specific standards of the World Health Organization [43]. In addition to the crude model, age, height and BMI were adjusted in model 1. On this basis, more confounding factors were added in model 2, including stage of puberty, physical activity, sedentary behavior, dietary energy intake, energy-adjusted calcium intake, and dietary vitamin D intake. The level of significance for all statistical tests was 0.05. All statistical analyses were performed using SPSS software version 23.0 for Windows (SPSS, Inc., Chicago, IL, USA.).

## 3. Results

### 3.1. Descriptive Characteristics

The characteristics of the 693 participants are shown in Table 1. The mean ages were 14.95, 13.20, and 15.93 years in the total sample, the prepubertal group, and the pubertal group, respectively. For the sample, the average BMI was 18.95 ± 3.22 kg/m^2^ and the mean physical activity value was 13.23 ± 9.30 MET h/d. The dietary energy intake in the pubertal group (2557 ± 705 kcal/d) was higher than that in the prepubertal group (2321 ± 839 kcal/d, *p* < 0.001). The pubertal groups (4.06 ± 1.96 h/day) had a higher average value of total time in sedentary behavior daily than the prepubertal group (3.66 ± 2.63 h/day, *p* = 0.005). There were no differences in the dietary intakes of calcium and vitamin D between the prepubertal and pubertal groups (*p* > 0.050). The mean values in the total sample were 438.96 ± 182.12 mg/d for dietary calcium intake and 1.94 ± 1.51 μg/d for dietary vitamin D intake. The total body FM% was higher in the prepubertal participants (15.38 ± 8.43 %) than in the pubertal participants (12.79 ± 5.82 %; *p* < 0.001). However, the FM in the trunk region was higher in the pubertal group (3.17 ± 2.97 kg) than the prepubertal group (2.67 ± 3.00 kg; *p* < 0.001). No significant differences were observed in the total body FM or the FM at the limb region between the two subgroups (*p* > 0.050 for both). The pubertal participants had a higher ratio of trunk-to-limb FM and a lower ratio of total body FM-to-lean mass than the prepubertal participants (*p* < 0.001). For bone parameters, the SOS value of the pubertal group (1552.00 ± 30.91) was larger than that of the prepubertal group (1547.03 ± 25.24, *p* < 0.001). BUA and SI were not significantly different between the two subgroups, although their values were higher in the pubertal group (*p* > 0.050).

### 3.2. Associations of FM, FM Distribution Variables with Bone Parameters in the Total Sample

Table 2 and Supplement Table S1 display the associations between the variables of FM, FM distribution, and bone parameters in the 693 male adolescents. In the crude model (Supplement Table S1), the variables of FM and FM distribution showed positive correlations with BUA (β = 0.118 to 0.170, *p* < 0.010), SI (β = 0.077 to 0.110, *p* < 0.050), and negative correlations with SOS (β = −0.076 to −0.091, *p* < 0.050) without adjusting the potential confounders. After the adjustment of age, height, and BMI in model 1 (Table 2), the variables of FM and FM distribution, except for the trunk-to-limb FM ratio, all showed significantly negative associations with SOS (β = −0.319 to −0.449, *p* < 0.001) and SI (β = −0.180 to −0.299, *p* ≤ 0.007). In model 2 (Table 2), all of those associations in model 1 remained significant (*p* ≤ 0.010) after the adjustment of age, height, BMI, stage of puberty, physical activity, sedentary behavior, dietary energy intake, energy-adjusted dietary calcium intake, and dietary vitamin D intake. SOS was negatively related to total body FM, total body FM%, trunk FM, limb FM, and total body FM-to-lean mass ratio (β = −0.319 to −0.454, *p* < 0.001) in model 2. Similarly, SI showed negative associations with total body FM, total body FM%, trunk FM, limb FM, and total body FM-to-lean mass ratio (β = −0.179 to −0.302, *p* < 0.010). BUA was inversely correlated with limb FM (β = −0.157, *p* = 0.043) in model 1 when adjusting for age, height and BMI. However, after further adjusting for stage of puberty, physical activity, sedentary behavior, dietary energy intake, energy-adjusted dietary calcium intake, and dietary vitamin D intake, the significance of the correlation disappeared (*p* = 0.067, model 2, Table 2). There were no significant relationships between BUA and total body FM, total body FM%, trunk FM, trunk-to-limb FM ratio, or total body FM-to-lean mass ratio in either model 1 or model 2 (all *p* > 0.050). The strongest relationship was between trunk FM and the bone parameter SOS.

### 3.3. Associations of FM, FM Distribution Variables with Bone Parameters in the Prepubertal Boys

Table 3 and Supplement Table S2 present the association between FM, FM distribution variables, and bone parameters in the prepubertal male participants (*n* = 246). In the crude model (Supplement Table S2), the variables of FM and FM distribution showed positive correlations with BUA (β = 0.135 to 0.140, *p* < 0.050) and negative correlations with SOS (β = −0.127 to −0.224, *p* < 0.050) without adjusting the potential confounders. After the adjustment of age, height, and BMI, SOS was negatively associated with total body FM, trunk FM, and limb FM (β = −0.363 to −0.374, *p* < 0.050, Table 3) in model 1. These associations of total body FM (β = −0.389, *p* = 0.019), trunk FM (β = −0.392, *p* = 0.028), and limb FM (β = −0.375, *p* = 0.009) with SOS remained significant in model 2 after adjusting for age, height, BMI, physical activity, sedentary behavior, dietary energy intake, energy-adjusted dietary calcium intake, and dietary vitamin D intake (Table 3). BUA and SI showed no correlation with FM or FM distribution variables both in model 1 and model 2 in prepubertal boys (*p* > 0.050, Table 3). Among the FM and FM distribution variables, trunk FM showed the strongest relationship with SOS in the prepubertal group, as in the total sample. The Supplement Figures S1–S3 also illustrated the relationships of the variables of FM and FM distribution with the studied bone parameters in the prepubertal boys.

### 3.4. Associations of FM, FM Distribution Variables with Bone Parameters in the Pubertal Boys

Table 4 and Supplement Table S3 show the association between FM, FM distribution variables, and bone parameters in the pubertal male participants (*n* = 447). In the crude model (Supplement Table S3), the variables of FM and FM distribution showed positive correlations with BUA (β = 0.141 to 0.208, *p* < 0.010) and SI (β = 0.102 to 0.143, *p* < 0.050) without adjusting the potential confounders. In this subgroup, after the adjustment of the confounders, all of the variables of FM and FM distribution, except for the trunk-to-limb FM ratio, had significant relationships with all of the bone parameters (i.e., BUA, SOS and SI) in both model 1 and model 2 (*p* < 0.050, Table 4). In model 2, after adjusting for age, height, BMI, physical activity, sedentary behavior, dietary energy intake, energy-adjusted dietary calcium intake, and dietary vitamin D intake, BUA was negatively associated with total body FM, total body FM%, trunk FM, limb FM, and total body FM-to-lean mass ratio (β = −0.204 to −0.330, *p* < 0.050). SOS was also inversely related to total body FM, total body FM%, trunk FM, limb FM, and total body FM-to-lean mass ratio (β = −0.322 to −0.479, *p* < 0.010) in model 2. Similarly, SI had negative associations with total body FM, total body FM%, trunk FM, limb FM, and total body FM-to-lean mass ratio (β = −0.282 to −0.427, *p* < 0.010) in model 2. Compared with the prepubertal group, the relationships of FM and FM distribution variables with bone parameters were stronger in the pubertal group. In addition, in the pubertal group, the strongest association was between trunk FM and SOS, consistent with the results in the total sample and in the prepubertal group. The Supplement Figures S1–S3 also illustrates the relationships of the variables of FM and FM distribution with the studied bone parameters in the pubertal boys.

## 4. Discussion

This study investigated the relationships of body fat and fat distribution variables with bone phenotypes in 693 Chinese male adolescents. FM and its distribution variables were significantly associated with the lower values of bone parameters (BUA, SOS, and SI) in Chinese boys after adjusting for important confounding factors. Among the FM-related variables, trunk FM had the strongest relationship with the bone parameter. This study also observed that the relationships of body fat and fat distribution variables with bone parameters in pubertal boys were stronger than those in prepubertal boys.

The results revealed that the total body and regional fat tissue (i.e., trunk and limb) all showed inverse associations with bone parameters (i.e., BUA, SOS, SI) in Chinese adolescent boys. Fat tissue at the trunk region had a stronger negative relationship with the studied bone parameter than did the other studied fat tissue-related variables. These findings are consistent with previous studies in children and adolescents [13,16,44,45]. Recently, Rokoff et al., in 876 children aged 6–10 years, also found that the inverse association between fat tissue and bone mass was driven by truncal rather than non-truncal (i.e., extremity) fat deposition [16]. Another longitudinal study of 350 teenagers aged 11–19 years found a negative association between visceral adipose tissue and cortical bone strength but no significant association for subcutaneous adipose tissue [46]. Adipose tissue is metabolically active and can secrete factors that affect bone metabolism. First, inflammatory factors such as interleukin-6 and tumor necrosis factor-α released by fat tissue at the trunk region may increase bone resorption and impede skeletal development by stimulating osteoclast activity [47]. For example, mice with high trunk FM simultaneously showed greater levels of inflammation and reduced bone mass [48]. Consistent with this, C-reactive protein, an inflammatory marker, was negatively associated with whole body BMD in American adults after adjusting for important confounders [49]. Second, adiponectin secreted by adipose tissue is negatively related to visceral fat accumulation and insulin resistance and thus could improve insulin sensitivity [46]. In addition, the secretion of free fatty acids by visceral adipose tissue could suppress the expression of insulin receptors, resulting in insulin resistance [16]. Animal studies have shown that insulin resistance can impair osteoblastic insulin signaling and affect osteoblast proliferation and survival, and consequently block bone formation [50]. Studies in adolescents have also found that those with higher insulin resistance had lower bone mass [51]. Therefore, greater trunk FM leading to lower BMD may be a consequence of inflammation and insulin resistance.

In this study, the association between adipose tissue and bone parameters was stronger in pubertal boys than in prepubertal boys. The variables of FM and its distribution were significantly related to all three studied bone parameters (BUA, SOS, and SI) in the pubertal group, but were only significantly associated with SOS and had smaller regression coefficient values in the prepubertal group. An observational study in 295 healthy children and adolescents aged 5–19 years showed that the percent of body fat had a significantly inverse correlation with bone mass in pubertal males and vice versa in individuals not attaining puberty [52]. Ackerman et al. [53] also reported a similar trend for the association between FM and bone mineral content in boys. Another 2-year cohort study of 1918 adolescent girls in Southwest England observed no association between total body FM and bone health in early pubertal girls, but observed a negative relationship between these parameters in Tanner stage 3 girls [54]. The possible explanations for this difference are as follows. Puberty is one of the most critical periods for general fat development and its distribution in adolescents. This can be explained by the differentiation of sex hormones’ concentration. In boys, testosterone concentration grows already in mid-childhood when there are no visible external indications of puberty and the differences in body composition become clear [55]. During puberty the circulating testosterone is responsible for the accumulation of trunk fat. Studies have shown that puberty and maturational events may increase the concentration of fat in the central region, leading to a more android shape in boys [21,56,57]. Maria et al. [58] concluded that body fat in boys is evenly distributed at the age of 13.45, but after it the dominance of trunk fat begins to increase gradually, based on 12-year (from 7 to 18 years old) longitudinal data of 270 boys from Cracow, Poland. In this study, the data also showed that the absolute FM of the trunk region and the ratio of trunk to limb FM in the pubertal group were higher than those in the prepubertal group, although no significant difference was observed in the total body FM between the two subgroups. This observed trend toward greater central fatness in the pubertal group could be due to a redistribution of subcutaneous fat from the extremities to the trunk during adolescence [59]. In addition, another potential explanation for the observed correlations might be the prolonged sedentary time. In this study, the pubertal groups had an increased sedentary behavior without changes in physical activity levels. Sedentary lifestyle patterns in children and adolescents have been found to be associated with obesity [60]. A 7-year follow-up study found that the accumulated sedentary time had a significantly positive association with total and abdominal FM. This relationship remained stable over the follow-up [61]. A study proposed that the increased sedentary time leads to a decreased skeletal muscle insulin sensitivity, which leads to redistribution of energy substrates into storage, contributing to central fat accumulation and ectopic storage in the liver and other organs [62]. As mentioned before, evidence indicates that central fat may be more deleterious to bone health [19,20,46]. Therefore, greater fat tissue accumulation at the trunk region in pubertal boys than in prepubertal boys may partly explain why the body fat and fat distribution measures had a stronger adverse association with bone health in the pubertal group. Studies have also suggested that bone mass accretion occurs more rapidly in late puberty than during prepuberty [63]. The negative association between adipose tissue and bone mass warrants closer attention, especially in pubertal individuals, as adolescents at this developmental stage may have the best opportunity to maximize their peak bone mass and decrease the risk of osteoporosis in later life.

To the best of our knowledge, this is the first study to reveal that fat tissue distribution variables has a stronger negative relationship with bone mass accumulation in Chinese pubertal boys than in prepubertal boys. However, this study also has some limitations. First, QUS and BIA were used to measure bone parameters and body composition, respectively, rather than the more accurate methods, i.e., DXA and MRI [25,64]. Nonetheless, these two measurements have been proven to be strongly correlated with those more accurate measures in pediatric population [26,29,31,64]. QUS and BIA are widely used in epidemiologic studies of children and adolescents for the merits of safety, easy-to-use, cost effectiveness and portability. In addition, BIA may underestimate the values of FM and percent body fat [65,66,67]. The results should be interpreted with caution. This systematic bias could underestimate the observed negative association between fat tissue and bone mass in adolescents in this study. Second, this study included male participants from only one secondary school in Jiangmen, China. Approximately 70% of the studied boys had a normal weight, which may have implied a healthy sample bias. In addition, the ethnical homogeneity in body composition was obvious, and body fat changes during childhood and adolescence are ethnic-related [22]. These all may restrict the generalization of the findings of this study. Third, a disproportionate difference existed in sample size between the two subgroups. The results in the overall group may have been mainly attributed to the results in the pubertal group with a much larger sample size. Hence, the negative association in the overall population could be overrated. Fourth, the cross-sectional design could not create cause–effect inference, and the influence of changes in body fat and fat distribution on bone mass accretion in adolescent boys could not be explored. Therefore, further investigations with a longitudinal design are warranted.

## 5. Conclusions

Adipose tissue was found to be a negative predictor of bone health in Chinese male adolescents. Among the fat parameters, fat tissue at the trunk region had the strongest negative association with bone density. Bone parameters were more sensitive to fat tissue in pubertal boys than in prepubertal boys. These findings highlight adipose tissue as a risk factor for pubertal bone formation in Chinese boys, and may provide useful information for the establishment of a prediction model for peak bone mass in Chinese male adolescents.

## Figures and Tables

**Table 1:** Basic characteristics of the studied subjects.

Variables	Total (*n* = 693)	Prepubertal (*n* = 246)	Pubertal (*n* = 447)	*p*
Age (years)	14.95 ± 1.45	13.20 ± 0.75	15.93 ± 0.53	**<0.001**
Height (m)	1.65 ± 0.11	1.56 ± 0.09	1.71 ± 0.06	**<0.001**
Weight (kg)	52.30 ± 12.31	43.94 ± 11.25	57.27 ± 10.01	**<0.001**
BMI (kg/m^2^)	18.95 ± 3.22	17.96 ± 3.27	19.63 ± 2.93	**<0.001**
Physical activity (MET·h/d)	13.23 ± 9.30	12.87 ± 8.65	13.42 ± 9.62	0.455
Sedentary behavior (h/d)	3.88 ± 2.23	3.66 ± 2.63	4.06 ± 1.96	**0.005**
Dietary energy intake (kcal/d)	2474 ± 767	2321 ± 839	2557 ± 705	**<0.001**
Dietary calcium intake (mg/d)	438.96 ± 182.12	436.07 ± 191.94	441.02 ± 176.06	0.732
Dietary vitamin D intake (ug/d)	1.94 ± 1.51	1.93 ± 1.56	1.95 ± 1.49	0.827
BUA (dB/MHz)	68.94 ± 15.92	68.11 ± 17.43	69.92 ± 18.41	0.208
SOS (m/s)	1548.89 ± 25.60	1547.03 ± 25.24	1552.00 ± 30.91	**<0.001**
SI	59.88 ± 15.64	58.80 ± 16.66	61.41 ± 19.23	0.074
Total body FM (kg)	7.53 ± 5.30	7.16 ± 5.52	7.73 ± 5.18	0.177
Total body FM% (%)	13.71 ± 6.97	15.38 ± 8.43	12.79 ± 5.82	**<0.001**
Trunk FM (kg)	2.99 ± 2.99	2.67 ± 3.00	3.17 ± 2.97	**<0.001**
Limb FM (kg)	3.66 ± 2.22	3.68 ± 2.41	3.65 ± 2.12	0.860
Trunk-to-limb FM ratio	0.68 ± 0.32	0.56 ± 0.33	0.74 ± 0.29	**<0.001**
Total body FM-to-lean mass ratio	0.31 ± 0.21	0.37 ± 0.28	0.27 ± 0.15	**<0.001**

Data are presented as mean ± standard deviation (SD). BMI, body mass index; BUA, broadband ultrasound attenuation; SOS, speed of sound; SI, stiffness index; FM, fat mass; bold: *p* < 0.05.

**Table 2:** The results of associations between FM, FM distribution variables, and bone parameters in the total sample (*n* = 693).

	BUA				SOS				SI			
	Model 1		Model 2		Model 1		Model 2		Model 1		Model 2	
	sβ	*p*	sβ	*p*	sβ	*p*	sβ	*p*	sβ	*p*	sβ	*p*
Total body FM (kg)	−0.162	0.069	−0.163	0.069	−0.434	**<0.001**	−0.437	**<0.001**	−0.299	**0.001**	−0.302	**0.001**
Total body FM% (%)	−0.062	0.385	−0.061	0.390	−0.342	**<0.001**	−0.340	**<0.001**	−0.192	**0.007**	−0.180	**0.008**
Trunk FM (kg)	−0.152	0.113	−0.152	0.114	−0.449	**<0.001**	−0.454	**<0.001**	−0.299	**0.002**	−0.302	**0.002**
Limb FM (kg)	−0.157	**0.043**	−0.144	0.067	−0.382	**<0.001**	−0.385	**<0.001**	−0.273	**<0.001**	−0.275	**<0.001**
Trunk-to-limb FM ratio	0.053	0.429	0.054	0.423	−0.088	0.192	−0.078	0.253	−0.004	0.948	−0.006	0.930
Total body FM-to-lean mass ratio	−0.058	0.381	−0.057	0.390	−0.319	**<0.001**	−0.319	**<0.001**	−0.180	**0.007**	−0.179	**0.007**

Model 1 represents the linear regression analyses with the adjustment of age, height, BMI; and model 2 represents model 1 + stage of puberty, physical activity, sedentary behavior, dietary energy intake, energy-adjusted dietary calcium intake, and dietary vitamin D intake. BUA, broadband ultrasound attenuation; SOS, speed of sound; SI, stiffness index; FM, fat mass; sβ, standardized regression coefficient; bold: *p* < 0.05.

**Table 3:** The results of associations between FM, FM distribution variables, and bone parameters in the prepubertal boys (*n* = 246).

	BUA				SOS				SI			
	Model 1		Model 2		Model 1		Model 2		Model 1		Model 2	
	sβ	*p*	sβ	*p*	sβ	*p*	sβ	*p*	sβ	*p*	sβ	*p*
Total body FM (kg)	0.150	0.364	0.150	0.372	−0.372	**0.023**	−0.389	**0.019**	−0.053	0.750	−0.060	0.721
Total body FM% (%)	0.184	0.156	0.194	0.140	−0.221	0.087	−0.226	0.083	0.035	0.787	0.040	0.762
Trunk FM (kg)	0.207	0.242	0.207	0.250	−0.374	**0.034**	−0.392	**0.028**	−0.013	0.940	−0.021	0.906
Limb FM (kg)	0.070	0.625	0.069	0.635	−0.363	**0.011**	−0.375	**0.009**	−0.105	0.469	−0.110	0.450
Trunk-to-limb FM ratio	0.122	0.271	0.135	0.232	0.052	0.635	0.060	0.594	0.108	0.334	0.120	0.290
Total body FM-to-lean mass ratio	0.138	0.270	0.143	0.256	−0.239	0.054	−0.243	0.053	−0.005	0.970	−0.002	0.985

Model 1 represents the linear regression analyses with the adjustment of age, height, BMI; and model 2 represents model 1 + physical activity, sedentary behavior, dietary energy intake, energy-adjusted dietary calcium intake, and dietary vitamin D intake. BUA, broadband ultrasound attenuation; SOS, speed of sound; SI, stiffness index; FM, fat mass; sβ, standardized regression coefficient; bold: *p* < 0.05.

**Table 4:** The results of associations between FM, FM distribution variables, and bone parameters in the pubertal boys (*n* = 447).

	BUA				SOS				SI			
	Model 1		Model 2		Model 1		Model 2		Model 1		Model 3	
	sβ	*p*	sβ	*p*	sβ	*p*	sβ	*p*	sβ	*p*	sβ	*p*
Total body FM (kg)	−0.310	**0.004**	−0.305	**0.004**	−0.427	**<0.001**	−0.431	**<0.001**	−0.391	**<0.001**	−0.390	**<0.001**
Total body FM% (%)	−0.209	**0.015**	−0.204	**0.018 **	−0.339	**<0.001**	−0.337	**<0.001**	−0.287	**0.001**	−0.283	**0.001**
Trunk FM (kg)	−0.336	**0.003**	−0.330	**0.004 **	−0.475	**<0.001**	−0.479	**<0.001**	−0.429	**<0.001**	−0.427	**<0.001**
Limb FM (kg)	−0.255	**0.006**	−0.251	**0.007**	−0.341	**<0.001**	−0.347	**<0.001**	−0.317	**0.001**	−0.317	**0.001**
Trunk-to-limb FM ratio	<0.001	1.000	0.002	0.976	−0.156	0.051	−0.145	0.075	−0.070	0.375	−0.064	0.429
Total body FM-to-lean mass ratio	−0.218	**0.010**	−0.214	**0.012**	−0.321	**<0.001**	−0.322	**<0.001**	−0.284	**0.001**	−0.282	**0.001**

Model 1 represents the linear regression analyses with the adjustment of age, height, BMI; model 2 represents model 1 + physical activity, sedentary behavior, dietary energy intake, energy-adjusted dietary calcium intake, and dietary vitamin D intake. BUA, broadband ultrasound attenuation; SOS, speed of sound; SI, stiffness index; FM, fat mass; sβ, standardized regression coefficient; bold: *p* < 0.05.

## Data Availability

Requests for data may be directed to the corresponding author and are subject to institutional data use agreements.

## Supplementary Materials

The following are available online at https://www.mdpi.com/article/10.3390/nu13072163/s1, Figure S1: Depicts the association results between the variables related to fat distribution and BUA in prepubertal group (A) and pubertal group (B), with the adjustment of age, height, body mass index, physical activity, sedentary behavior, dietary energy intake, energy-adjusted dietary calcium intake and dietary vitamin D intake. (a): total body fat mass (FM, kg); (b): total body FM% (%); (c): trunk fat (kg); (d): limb fat (kg); (e): trunk to limb FM ratio; (f): total body FM to lean mass (LM) ratio. BUA, broadband ultrasound attenuation. Figure S2: Depicts the association results between the variables related to fat distribution and SOS in prepubertal group (A) and pubertal group (B), with the adjustment of age, height, body mass index, physical activity, sedentary behavior, dietary energy intake, energy-adjusted dietary calcium intake and dietary vitamin D intake. (a): total body fat mass (FM, kg); (b): total body FM% (%); (c): trunk fat (kg); (d): limb fat (kg); (e): trunk to limb FM ratio; (f): total body FM to lean mass (LM) ratio. SOS, speed of sound. Figure S3: Depicts the association results between the variables related to fat distribution and SI in prepubertal group (A) and pubertal group (B), with the adjustment of age, height, body mass index, physical activity, sedentary behavior, dietary energy intake, energy-adjusted dietary calcium intake and dietary vitamin D intake. (a): total body fat mass (FM, kg); (b): total body FM% (%); (c): trunk fat (kg); (d): limb fat (kg); (e): trunk to limb FM ratio; (f): total body FM to lean mass (LM) ratio. SI, stiffness index. Table S1: The results of associations between FM, FM distribution variables and bone parameters in the total sample (*n* = 693). Table S2: The results of associations between FM, FM distribution variables and bone parameters in the prepubertal boys (*n* = 246). Table S3: The results of associations between FM, FM distribution variables and bone parameters in the pubertal boys (*n* = 447).
