# Supplementary material for: Analysis of the Association between Fat Mass Distribution and Bone Mass in Chinese Male Adolescents at Different Stages of Puberty
Source: Nutrients. 2021 Jun 24;13(7):2163. doi: 10.3390/nu13072163 (PMC8308367; doi:10.3390/nu13072163)
Supplement: Supplementary file 1 [file nutrients-13-02163-s001.zip › nutrients-1275854-supplementary.pdf]

Article

Analysis of the Association between Fat Mass Distribution and Bone Mass in Chinese Male Adolescents at Different Stage of Puberty

Kai-Li Deng [1], Hui-Li [1], Wan-Yu Yang [1], Jin-Li Hou [1], Yang Xu [1] and Su-Mei Xiao [1,2,*]

[1] Department of Epidemiology, School of Public Health, Sun Yat-sen University, Guangzhou, Guangdong, China; dengkli@mail2.sysu.edu.cn

[2] Guangdong Provincial Key Laboratory of Food, Nutrition and Health, School of Public Health, Sun Yat-sen University; xiaosm3@mail.sysu.edu.cn

* Correspondence: xiaosm3@mail.sysu.edu.cn; Tel.: +86-20-8733-0151

Supplement Table S1. The results of associations between FM, FM distribution variables and bone parameters in the total sample (n=693)

The results represented the univariate analyses between the variables related to fat distribution and bone parameters. BUA, broadband ultrasound attenuation; SOS, speed of sound; SI, stiffness index; FM, fat mass; sβ, standardized regression coefficient; bold: $p < 0.05$.

	BUA		SOS		SI	
	sβ	*p*	sβ	*p*	sβ	*p*
Total body FM (kg)	0.144	**<0.001**	-0.062	0.105	0.068	0.074
Total body FM% (%)	0.130	**0.001**	-0.084	**0.027**	0.048	0.205
Trunk FM (kg)	0.152	**<0.001**	-0.053	0.160	0.077	**0.044**
Limb FM (kg)	0.130	**0.001**	-0.076	**0.045**	0.052	0.175
Trunk to limb FM ratio	0.170	**<0.001**	-0.005	0.899	0.110	**0.004**
Total body FM to lean mass ratio	0.118	**0.002**	-0.091	**0.016**	0.037	0.327

Supplement Table S2. The results of associations between FM, FM distribution variables and bone parameters in the prepubertal boys (n=246)

	BUA		SOS		SI	
	sβ	*p*	sβ	*p*	sβ	*p*
Total body FM (kg)	0.086	0.176	-0.215	**0.001**	-0.031	0.630
Total body FM% (%)	0.140	**0.027**	-0.180	**0.005**	0.022	0.728
Trunk FM (kg)	0.087	0.171	-0.211	**0.001**	-0.028	0.659
Limb FM (kg)	0.082	0.200	-0.224	**<0.001**	-0.038	0.554
Trunk to limb FM ratio	0.088	0.169	-0.127	**0.046**	0.008	0.906
Total body FM to lean mass ratio	0.135	**0.034**	-0.179	**0.005**	0.019	0.768

The results represented the univariate analyses between the variables related to fat distribution and bone parameters. BUA, broadband ultrasound attenuation; SOS, speed of sound; SI, stiffness index; FM, fat mass; sβ, standardized regression coefficient; bold: $p < 0.05$.

Supplement Table S3. The results of associations between FM, FM distribution variables and bone parameters in the pubertal boys (n=447)

	BUA		SOS		SI	
	sβ	*p*	sβ	*p*	sβ	*p*
Total body FM (kg)	0.174	**<0.001**	0.005	0.918	0.114	**0.016**
Total body FM% (%)	0.146	**0.002**	-0.004	0.930	0.092	0.052
Trunk FM (kg)	0.182	**<0.001**	0.008	0.864	0.120	**0.011**
Limb FM (kg)	0.160	**0.001**	-0.001	0.977	0.102	**0.032**
Trunk to limb FM ratio	0.208	**<0.001**	0.021	0.664	0.143	**0.003**
Total body FM to lean mass ratio	0.141	**0.003**	-0.002	0.962	0.090	0.058

The results represented the univariate analyses between the variables related to fat distribution and bone parameters. BUA, broadband ultrasound attenuation; SOS, speed of sound; SI, stiffness index; FM, fat mass; sβ, standardized regression coefficient; bold: $p < 0.05$.

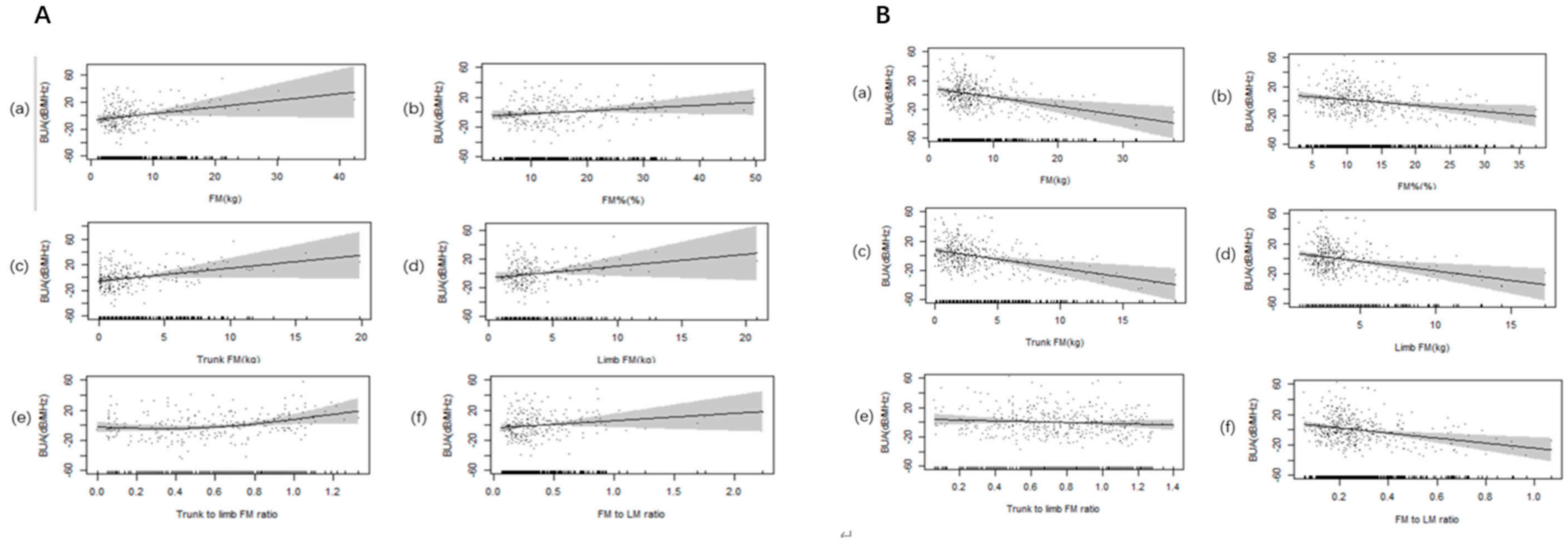

Supplement Figure S1 depicts the association results between the variables related to fat distribution and BUA in prepubertal group (A) and pubertal group (B), with the adjustment of age, height, body mass index, physical activity, sedentary behavior, dietary energy intake, energy-adjusted dietary calcium intake and dietary vitamin D intake. (a): total body fat mass (FM, kg); (b): total body FM% (%); (c): trunk fat (kg); (d): limb fat (kg); (e): trunk to limb FM ratio; (f): total body FM to lean mass (LM) ratio. BUA, broadband ultrasound attenuation.

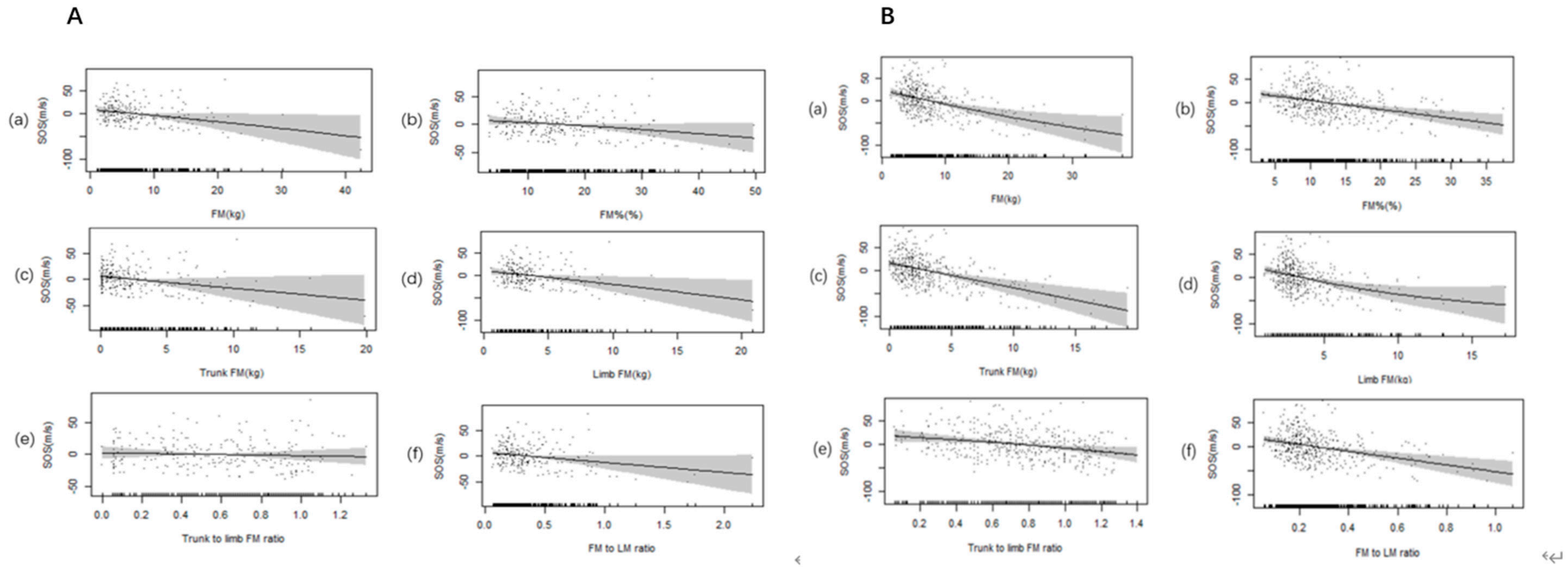

Supplement Figure S2 depicts the association results between the variables related to fat distribution and SOS in prepubertal group (A) and pubertal group (B), with the adjustment of age, height, body mass index, physical activity, sedentary behavior, dietary energy intake, energy-adjusted dietary calcium intake and dietary vitamin D intake. (a): total body fat mass (FM, kg); (b): total body FM% (%); (c): trunk fat (kg); (d): limb fat (kg); (e): trunk to limb FM ratio; (f): total body FM to lean mass (LM) ratio. SOS, speed of sound.

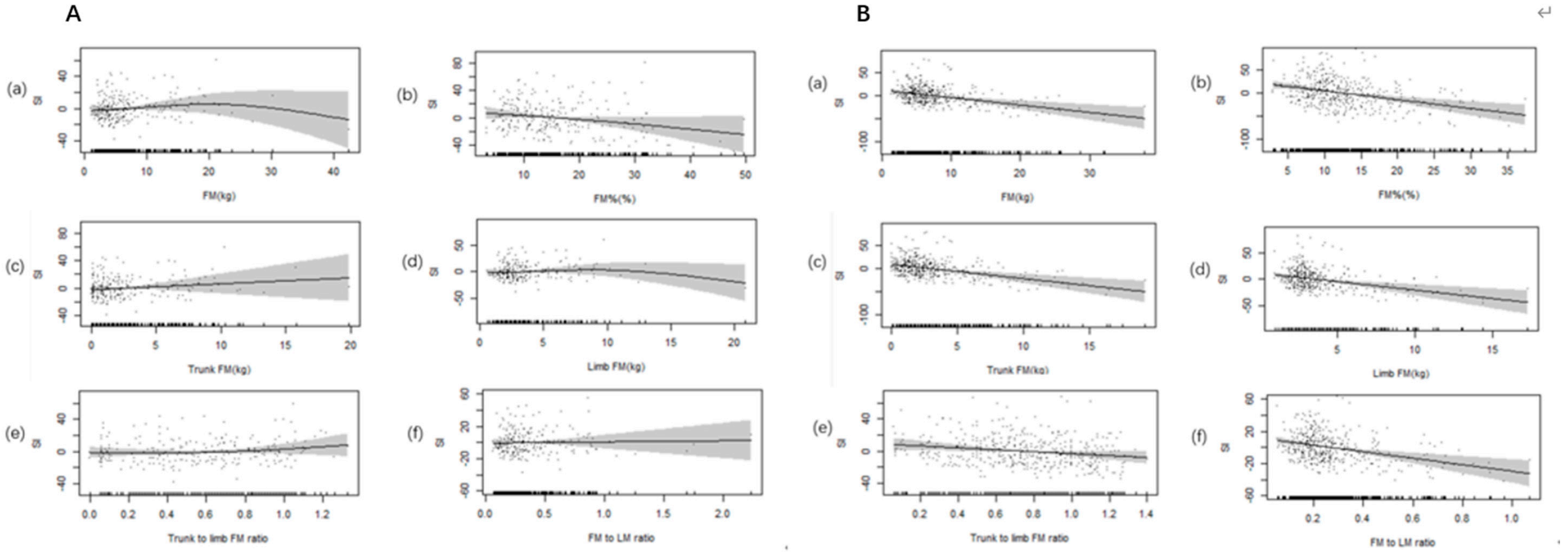

Supplement Figure S3 depicts the association results between the variables related to fat distribution and SI in prepubertal group (A) and pubertal group (B), with the adjustment of age, height, body mass index, physical activity, sedentary behavior, dietary energy intake, energy-adjusted dietary calcium intake and dietary vitamin D intake. (a): total body fat mass (FM, kg); (b): total body FM% (%); (c): trunk fat (kg); (d): limb fat (kg); (e): trunk to limb FM ratio; (f): total body FM to lean mass (LM) ratio. SI, stiffness index.
